# Prognostic impact of lingual lymph node metastasis in patients with squamous cell carcinoma of the tongue: a retrospective study

**DOI:** 10.1038/s41598-021-99925-2

**Published:** 2021-10-15

**Authors:** Takeshi Kuroshima, Yusuke Onozato, Yu Oikawa, Toshimitsu Ohsako, Takuma Kugimoto, Hideaki Hirai, Hirofumi Tomioka, Yasuyuki Michi, Masahiko Miura, Ryoichi Yoshimura, Hiroyuki Harada

**Affiliations:** 1 Division of Health Science, Department of Oral and Maxillofacial Surgery, Graduate School of Medical and Dental Sciences, Tokyo Medical and Dental University, Bunkyo-ku, 1-5-45 Yushima, Bunkyo-ku, Tokyo, 113-8549 Japan; 2 Division of Oral Health Science, Department of Oral Radiation Oncology, Graduate School of Medical and Dental Sciences, Tokyo Medical and Dental University, Bunkyo-ku, Tokyo, 113-8549 Japan; 3 Division of Maxillofacial and Neck Reconstruction, Department of Radiation Therapeutics and Oncology, Graduate School of Medical and Dental Sciences, Tokyo Medical and Dental University, Bunkyo-ku, Tokyo, 113-8549 Japan

**Keywords:** Cancer, Metastasis, Oral cancer

## Abstract

Squamous cell carcinoma (SCC) of the tongue rarely metastasizes to the lingual lymph nodes (LLNs), which are inconstant nodes and often situated outside the areas of basic tongue tumor surgery. The current study evaluated the clinicopathological features and prognostic impact of LLN metastasis (LLNM), compared to that of cervical lymph node metastasis, in patients with tongue SCC. A total of 608 patients underwent radical surgery for tongue SCC at our department between January 2001 and December 2016. During neck dissection, we scrutinized and resected lateral LLNs, when present. Of the 128 patients with lymph node metastasis, 107 had cervical lymph node metastasis and 21 had both cervical lymph node metastasis and LLNM. Univariate analysis demonstrated that LLNM was significantly associated with the adverse features of cervical lymph node metastasis. The 5-year disease-specific survival (5y-DSS) was significantly lower in patients with LLNMs than in those without LLNMs (49.0% vs. 88.4%, P < 0.01). Moreover, Cox proportional hazards model analyses revealed that cervical lymph node metastasis at level IV or V and LLNM were independent prognostic factors for 5y-DSS. LLNM has a strong negative impact on survival in patients with tongue SCC. An advanced status of cervical lymph node metastasis may predict LLNM.

## Introduction

The oral cavity is the most common subsite of head and neck cancers^1^. More than 90% of malignancies in the oral cavity are squamous cell carcinomas (SCCs)^1,2^, and SCCs of the tongue and floor of the mouth account for > 50% of primary oral SCCs^3,4^. Despite progress in diagnosis and treatment, survival in patients with advanced oral SCC has not improved significantly^5,6^. Metastasis to the cervical lymph nodes is one of the most accurate prognostic factors in patients with oral SCC^1–3^. SCC arising in the tongue frequently metastasizes to the cervical lymph nodes compared to SCC arising at other subsites. However, tongue SCCs rarely metastasize to the lingual lymph nodes (LLNs) that interrupt the lymphatic collecting trunks draining from the tongue and floor of the mouth to the cervical lymph nodes^7^.

LLNs can be divided into two groups, median and lateral LLNs^7–10^. The median LLNs are situated in the lingual septum between the genioglossus and geniohyoid muscles^7–10^, whereas lateral LLNs are situated along the course of the lingual artery on the external surface of the genioglossus or hyoglossus muscle^7–10^. Furthermore, the lateral LLNs can divided into two groups: the parahyoid nodes, which are located along the course of the lingual artery at the cornu of the hyoid bone, and the paraglandular nodes, which are located in proximity to the sublingual gland^10^. LLNs are often not detected in any imaging examinations as they are frequently absent or are small when present^8,10–12^. Moreover, due to their anatomic locations, LLNs are often situated outside the areas of surgical resection of the primary tongue tumor and neck dissection^8,10,13–17^. For instance, the median LLNs cannot be resected during partial glossectomy that does not include the lingual septum. Lateral LLNs in the sublingual space cannot be resected during partial glossectomy that frequently does not include this space or during discontinuous neck dissection without scrutiny. If not investigated carefully, lateral LLNs in the parahyoid area may be overlooked during any type of neck dissection. Previous studies have reported the incidence of median and lateral lingual lymph node metastasis (LLNM) to be 0.7–3.0%^8,11,13^ and 1.4–14.3%^11,13,17–19^, respectively. However, there are insufficient data on the clinical implications and prognostic value of LLNM in patients with tongue SCC because LLNM has received little attention until recently.

We hypothesized that LLNM, similar to cervical lymph node metastasis, contributes to poor prognosis in patients with tongue SCC. The present study aimed to evaluate the clinicopathological features associated with LLNM and impact of LLNM, compared to that of cervical lymph node metastasis, on survival in patients with tongue SCC.

## Results

### Characteristics of patients and LLNM

Of the 608 patients who underwent treatment for tongue SCC during the study period, 128 (21.1%) had cervical lymph node metastasis or LLNM. These patients included 92 men and 36 women aged 21 to 83 (median, 60.5) years. Eighty-nine patients initially underwent both glossectomy and neck dissection; of these, 80 patients underwent continuous neck dissection with a pull-through maneuver and nine underwent discontinuous neck dissection. Thirty-nine patients initially underwent resection of the primary tumor alone, followed by neck dissection for delayed lymph node metastasis.

**Figure 1:**
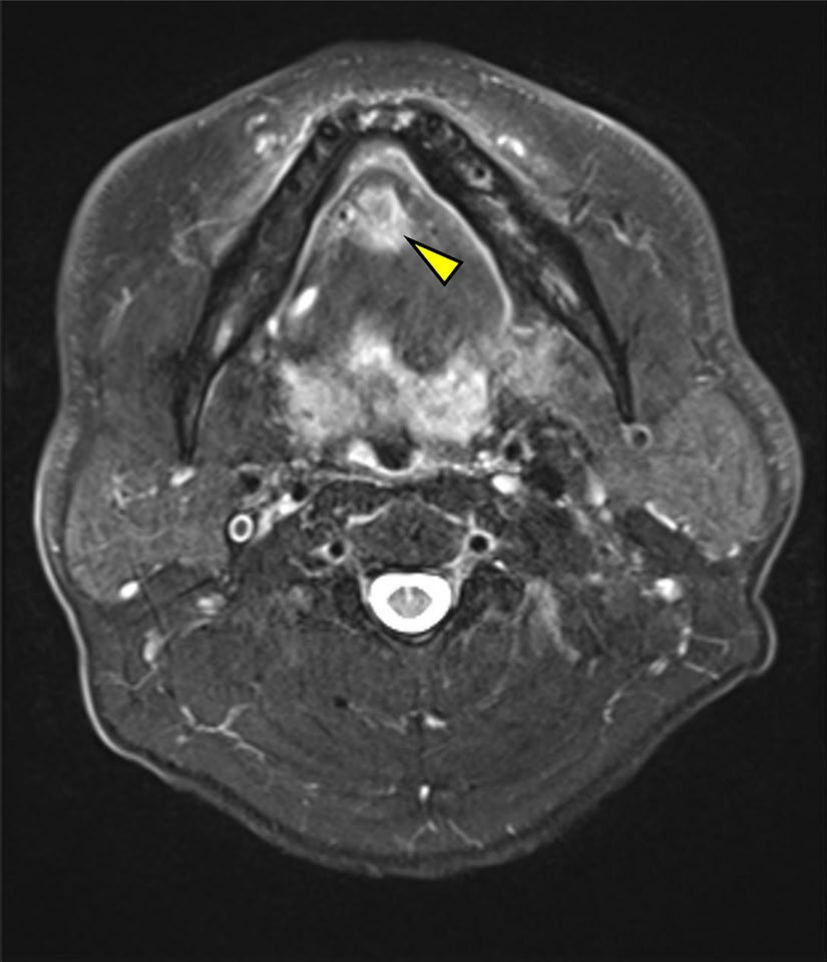
T2-weighted magnetic resonance (MR) image showing median lingual lymph node metastasis (LLNM) (arrowhead) in the lingual septum.

**Figure 2:**
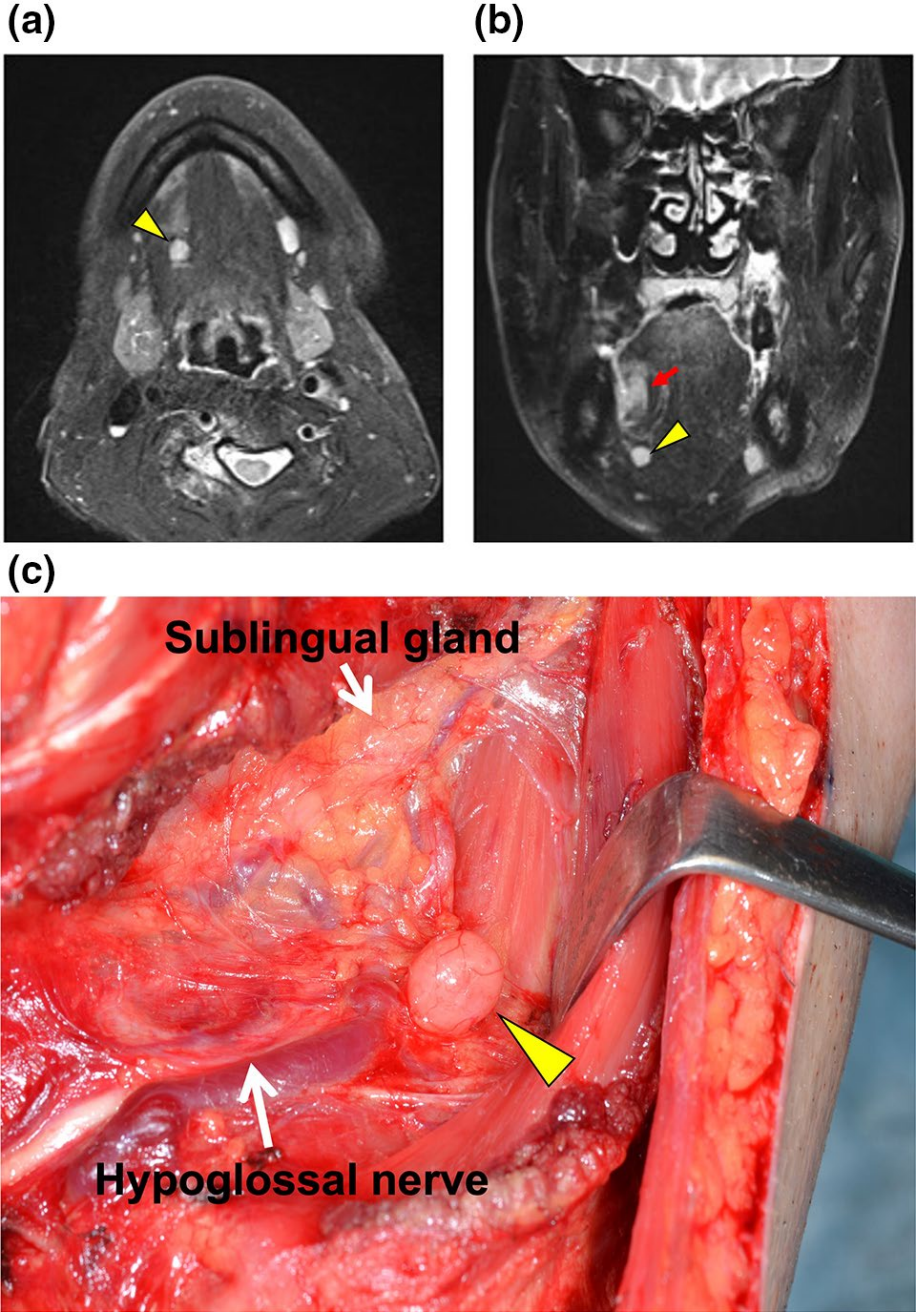
Imaging of lateral lingual lymph node metastasis (LLNM) in the sublingual space. Lateral LLNM (arrowhead) is revealed in the sublingual space on the right side on (**a**) axial T2-weighted magnetic resonance (MR) and (**b**) coronal T2-weighted MR images. This metastatic node is independent of the primary tongue tumor (arrow). (**c**) Lateral LLNM (arrowhead) is located in the proximity of the sublingual gland. The mylohyoid muscle is retracted anteriorly.

**Figure 3:**
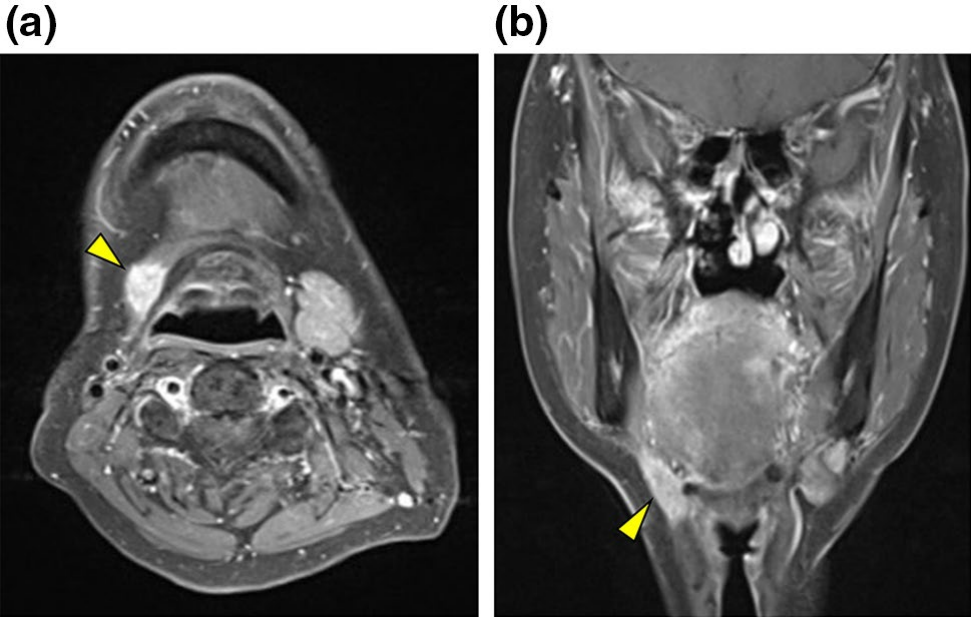
Imaging of lateral lingual lymph node metastasis (LLNM) in the parahyoid area. Lateral LLNM (arrowhead) is demonstrated in the parahyoid area on the right side on (**a**) enhanced axial T1-weighted magnetic resonance (MR) and (**b**) enhanced coronal T1-weighted MR images.

**Figure 4:**
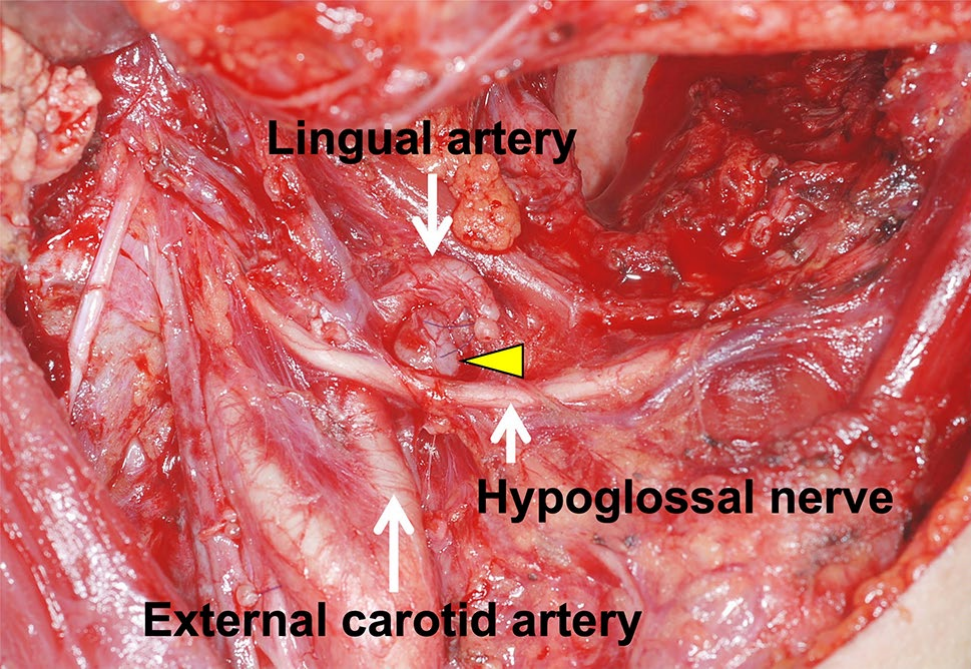
Imaging of subclinical lateral lingual lymph node metastasis in the parahyoid area. Supraomohyoid neck dissection and hemiglossectomy with a pull-through maneuver are performed. The lingual artery is ligated. Lateral lingual lymph node (arrowhead) is revealed below the lingual artery. This node is not shown in preoperative imaging.

Of the 128 patients, 107 had cervical lymph node metastasis without LLNM and 21 had both cervical lymph node metastasis and LLNM. Therefore, LLNM was detected in 3.5% of 608 patients with tongue SCC who underwent radical surgery during the study period. Of the 21 patients with LLNM, three had median LLNM (Fig. 1), seven had lateral LLNM in the sublingual space (Fig. 2), and 11 had lateral LLNM in the parahyoid area (Figs. 3 and 4). LLNM was confirmed during the first surgery in 14 patients, all of whom underwent resection of the primary tumor and neck dissection—12 patients underwent continuous neck dissection with a pull-through maneuver and two patients underwent discontinuous neck dissection. Among these 14 patients, LLNM was not detected in pretreatment examinations in 12 (85.7%) patients. Occult LLNM was evident in seven patients who subsequently developed cervical lymph node metastasis. Taken together, 90.5% (19/21) of the LLNMs were subclinical metastases. The median number of LLNM was one (range, 1 to 3). The median length of the long axis of the 12 subclinical LLNMs, which were proven at the first surgery, was 7.5 (range, 1 to 18) mm. Extranodal extension (ENE) of LLNMs was observed in 15 (71.4%) of the 21 patients.

### Correlation between clinicopathological features and LLNM

Univariate analyses were performed to compare clinicopathological features between patients with and without LLNM (Table 1). Age, sex, cT stage, and pathological differentiation were not significantly associated with LLNM. However, LLNM was significantly associated with the number (≤ 3 vs. ≥ 4), level (I–III vs. IV–V), and ENE (negative vs. positive) of ipsilateral cervical lymph node metastases as well as with involvement on the contralateral side (absence vs. presence).

**Table 1:** Univariate analyses of correlation between clinicopathological features and LLNM in tongue SCC patients with lymph node metastases.

Clinicopathological features	Cervical LNM without LLNM (n = 107)	Cervical LNM and LLNM (n = 21)	P-value
**Age**			0.58*
< 60 years	49	11	
> 60 years	58	10	
**Sex**			0.63*
Male	76	16	
Female	31	5	
**cT**			0.23*
T1 + T2	80	13	
T3 + T4	27	8	
**Pathological differentiation**			0.72*
Well	41	7	
Moderate	36	9	
Poor	26	5	
Unknown	4	0	
**Number of ipsilateral positive nodes**			< 0.01*
< 3	89	11	
≥ 4	18	10	
**Level of ipsilateral positive nodes**			0.02**
Level I, II, or III	103	17	
Level IV or V	4	4	
**ENE of ipsilateral positive nodes**			0.02*
Negative	60	6	
Positive	47	15	
**Contralateral cervical LNM**			< 0.01*
Absence	98	15	
Presence	9	6	

*SCC* squamous cell carcinoma, *LNM* lymph node metastasis, *LLNM* lingual lymph node metastasis, *ENE* extranodal extension.

*Chi-square test.

**Fisher’s exact test. P < 0.05 is considered statistically significant in all analyses.

### Survival rates in patients with and without LLNM

**Figure 5:**
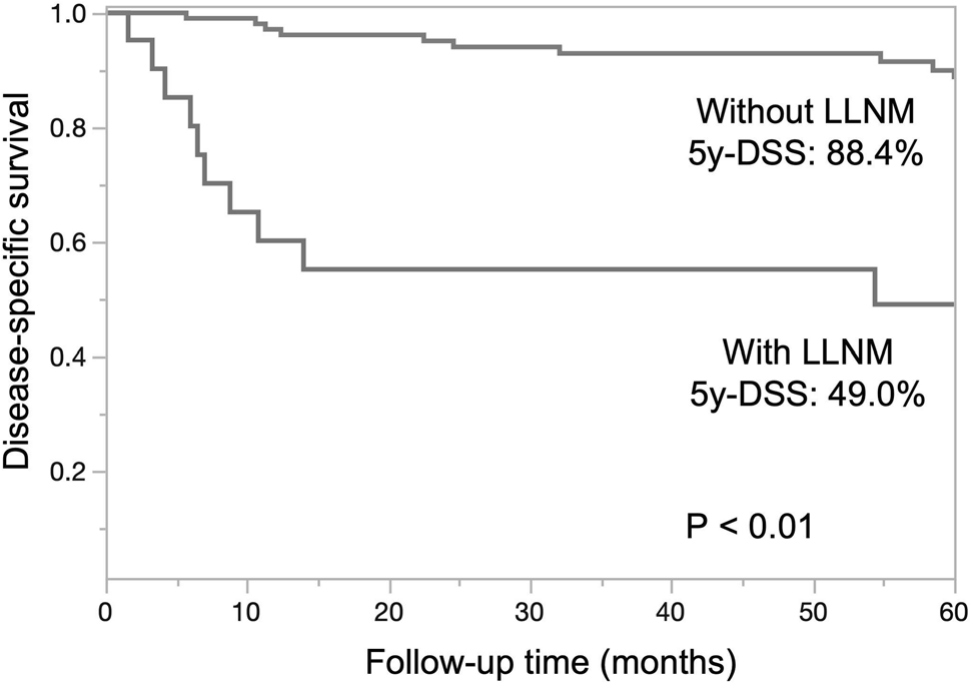
Kaplan–Meier survival curves and log-rank tests comparing 5-year disease-specific survival (5y-DSS) in patients with lingual lymph node metastases (LLNM) and patients without LLNM.

Univariate analyses of the factors related to 5-year disease-specific survival (5y-DSS) were performed. As shown in Table 2, the number of ipsilateral positive nodes, level of ipsilateral positive nodes, contralateral cervical lymph node metastasis, LLNM, and postoperative treatment were significantly associated with 5y-DSS. The 5y-DSS rates differed significantly between patients with and without LLNM (49.0% vs. 88.4%, P < 0.01; Fig. 5). Age, sex, cT stage, and pathological differentiation were not significantly associated with 5y-DSS. Cox proportional hazards model analysis demonstrated that level IV or V involvement of the ipsilateral positive nodes (hazard ratio [HR]: 12.46; 95% confidence interval [CI] 3.17–50.45; P < 0.01) and the presence of LLNM (HR 5.87; 95% CI 2.09–17.09; P < 0.01) were independent prognostic factors for 5y-DSS (Table 3).

**Table 2:** Univariate analyses of factors related to 5-year disease-specific survival in tongue SCC patients with lymph node metastases.

Clinicopathological factors	n	5y-DSS (%)	P-value
**Age**			0.49
< 60 years	60	84.9	
> 60 years	68	79.3	
**Sex**			0.48
Male	92	79.9	
Female	36	87.7	
**cT**			0.09
T1 + T2	93	85.3	
T3 + T4	35	71.7	
**Pathological differentiation**			0.53
Well	48	78.6	
Moderate	45	87.4	
Poor	28	76.6	
Unknown	4	100	
**Number of ipsilateral positive nodes**			< 0.01
< 3	100	88.8	
≥ 4	28	55.4	
**Level of ipsilateral positive nodes**			< 0.01
Level I, II, or III	120	86.8	
Level IV or V	8	12.5	
**ENE of ipsilateral positive nodes**			0.02
Negative	66	88.9	
Positive	62	74.4	
**Contralateral cervical LNM**			< 0.01
Absence	113	86.4	
Presence	15	46.9	
**LLNM**			< 0.01
Absence	107	88.4	
Presence	21	49.0	
**Postoperative treatment**			< 0.01
No	75	89.6	
Yes	53	71.3	

*SCC* squamous cell carcinoma, *5y-DSS* 5-year disease-specific survival, *LNM* lymph node metastasis, *LLNM* lingual lymph node metastasis, *ENE* extranodal extension.

Survival between patients is compared using the log-rank test.

P < 0.05 is considered statistically significant.

**Table 3:** Multivariate analyses of factors related to 5-year disease-specific survival in tongue SCC patients with lymph node metastasis.

Clinicopathological factors	Hazard ratio	P-value	95% CI
Number of ipsilateral positive nodes (≥ 4 vs. ≤ 3)	1.83	0.38	0.48–8.20
Level of ipsilateral positive nodes (Level IV or V vs. Level I, II, or III)	12.46	< 0.01	3.17–50.45
ENE of ipsilateral positive nodes (Positive vs. Negative)	1.66	0.36	0.56–5.23
Contralateral cervical LNM (Presence vs. Absence)	2.19	0.19	0.67–6.49
LLNM (Presence vs. Absence)	5.87	< 0.01	2.09–17.09
Postoperative treatment (Positive vs. Negative)	1.13	0.86	0.29–4.89

Analyses performed using Cox proportional hazards model.

*SCC* squamous cell carcinoma, *CI* confidence interval, *LNM* lymph node metastasis, *LLNM* lingual lymph node metastasis, *ENE* extranodal extension.

P < 0.05 is considered statistically significant.

## Discussion

The key findings of the present study were as follows: (1) The incidence of LLNM was 3.5% in 608 patients with tongue SCC. (2) All patients with LLNM also had cervical lymph node metastasis. Statistical analysis of the correlation between LLNM and clinicopathological features suggested that pathologic adverse features of cervical lymph node metastasis may be a reliable predictor of LLNM; (3) The 5y-DSS in patients with LLNM was significantly poorer than that in patients without LLNM. Multivariate analysis revealed that LLNM and level IV or V involvement of ipsilateral positive nodes were strong prognostic factors in patients with tongue SCC.

Few studies have investigated the clinicopathological factors related to LLNM in patients with tongue SCC. Jia et al. reported that among 111 patients with tongue SCC, five had LLNM and cervical lymph node status of pN2^17^. Moreover, T stage and occult cervical lymph node metastasis were associated with LLNM in patients with cT1-2N0 tongue SCC^18^, whereas T stage, tumor differentiation, perineural invasion, lymphovascular invasion, and cervical lymph node metastasis were associated with LLNM in patients with cT2-4 tongue SCC^19^. To the best of our knowledge, the present study is the first to statistically evaluate the clinicopathological features related to LLNM in all stages (cT1-4) of tongue SCC. We found that all patients with LLNM also had other cervical lymph node metastases. Univariate analysis showed that LLNM was significantly associated with the status of cervical lymph node metastasis, including ≥ 4 positive nodes, level IV or V involvement, ENE, and spread to the cervical lymph nodes on the contralateral side. These results suggest that cervical lymph node metastasis, especially advanced status, may predict LLNM in patients with tongue SCC. In contrast, there was no significant relationship between cT stage and LLNM, indicating that early T-stage tongue SCC can metastasize to the LLN.

Further, few studies have assessed the prognostic significance of LLNM in patients with tongue SCC. Yang et al. reported LLNM to be an independent prognostic factor for survival only in patients with cT1-2N0 tongue SCC, with a 5y-DSS of 51%^18^. Ando et al. reported that recurrent disease in the parahyoid area contributed to poor disease-specific survival (DSS) in 77 patients with regional failure of cT1-2 tongue SCC^14^. To the best of our knowledge, the present study is the first to assess the impact of LLNM on survival in patients at all stages (cT1-4) of tongue SCC. We evaluated the impact of LLNM on survival in patients with tongue SCC and compared it with that of cervical lymph node metastasis. The results indicated a significantly lower 5y-DSS rate in patients with LLNM than in those without LLNM (49.0% vs. 88.4%, P < 0.01). Furthermore, Cox proportional hazards analysis revealed that the number of ipsilateral positive nodes, ENE of ipsilateral positive nodes, and contralateral cervical lymph node metastasis were not associated with 5y-DSS. However, level IV or V involvement and LLNM were significantly associated with poor 5y-DSS. These results suggest that LLNM has a greater negative impact on survival in patients with tongue SCC than other representative adverse features of cervical lymph node metastasis.

In this study, most LLNMs were subclinical metastases, consistent with the results of previous studies^11,12,14,17^. To completely dissect subclinical median and lateral LLNMs, glossectomy including the tongue septum and continuous neck dissection using a pull-through maneuver are required. However, this surgical procedure may not be recommended uniformly in all patients with tongue SCC due to the low incidence of LLNM (3.5% in the present study). Therefore, it may be appropriate to carefully scrutinize and resect LLNs during any type of neck dissection. To scrutinize and resect the lateral LLN in the parahyoid area, we resected the digastric and stylohyoid muscles, which facilitated an adequate approach to this area with a clear visual field. We firstly scrutinized the lateral LLN along the hypoglossal nerve on the external surface of hyoglossus muscles. We next scrutinized the lateral LLN with a careful observation and palpation along the course of the lingual artery from the anterior surface of the external carotid artery to the hyoglossus muscle (Fig. 4). To avoid a swallowing dysfunction, the posterior belly of digastric muscle and stylohyoid muscle were spared on one side during bilateral neck dissection. Then, we retracted these muscles superiorly during lateral LLN scrutiny. A few reports also had recommended an inspection and dissection of the lateral LLN in the parahyoid area during neck dissection^14,15,17^. Regarding the lateral LLN in the sublingual space, we retracted the mylohyoid muscle anteriorly and scrutinized these nodes with careful palpation. Our procedure for the lateral LLN did not provide additional comorbidity with neck dissection, which is consistent with the findings of previous reports^14,15^. In addition to, careful surveillance for LLNM should be strictly performed in patients with tongue SCC.

This study has several limitations. First, as only few tongue SCC patients with cervical lymph node metastases and LLNM were included, the statistical power to draw firm conclusions was insufficient. Second, the survival benefits of scrutinizing LLNs during neck dissection and hemiglossectomy could not be evaluated due to the retrospective study design.

In conclusion, LLNMs, which rarely develop in patients with tongue SCC, are associated with poor survival outcomes. Most metastatic LLNs are subclinical and undetectable by imaging modalities before surgery. Therefore, LLNs should be carefully scrutinized and resected during neck dissection. Careful surveillance for LLNM is necessary in patients with tongue SCC. The adverse features of cervical lymph node metastases may be predictive of LLNMs. Additional large prospective studies are needed to verify the results of this study and establish the most appropriate treatment for LLNMs.

## Methods

### Patients

The medical records of 608 patients with tongue SCC who underwent radical surgery at the Department of Oral Maxillofacial Surgery at Tokyo Medical and Dental University Hospital between January 2001 and December 2016 were retrospectively reviewed. Patients who received previous treatments for tongue tumors were excluded. Data on patients with pathologically confirmed cervical lymph node metastasis or LLNM were analyzed. Demographic and clinical data, including age, sex, T stage, pathological differentiation of the primary tumor, mode of cervical lymph node metastasis, and treatment outcomes, were obtained from patients’ medical records. Tumors were clinically staged according to the 7th edition of the American Joint Committee on Cancer TNM staging system^20^. LLNs were divided into three groups: median LLNs (located in the lingual septum between the genioglossus and geniohyoid muscles on both sides), lateral LLNs in the sublingual space (located along the genioglossus or hyoglossus muscle), and lateral LLNs in the parahyoid area (located along the course of the lingual artery or hypoglossal nerve at the cornu of the hyoid bone)^7–10^. This study complied with the Declaration of Helsinki and was approved by the Institutional Review Board of Tokyo Medical and Dental University (D2015-600). Informed consent was obtained in the form of opt-out on the in-hospital bulletin. Patients who rejected participation in this study were excluded.

### Treatments

All patients underwent preoperative imaging, including computed tomography (CT), magnetic resonance imaging (MRI), ultrasonography, and ^18^F-fluorodeoxyglucose positron emission tomography/computed tomography (FDG-PET/CT). The primary tumor and cervical lymph node metastases were assessed based on physical examination and imaging findings. All primary tumors were resected with surgical margins ≥ 10 mm. We followed a wait-and-watch strategy for the management of clinically negative cervical lymph node metastases (cN0) in patients with tongue SCC. When patients with cN0 underwent excision of the primary tumor and reconstruction using a vascularized free flap, supraomohyoid neck dissection (levels I, II, and III) was performed as elective neck dissection. Patients with clinically positive cervical lymph nodes underwent radical or modified radical neck dissection at levels I–V.

During any neck dissection, we resected the digastric and stylohyoid muscles and scrutinized the lateral LLN along the course of the lingual artery and hypoglossal nerve at the cornu of the hyoid bone. When the mylohyoid muscle was spared, we anteriorly retracted this muscle and scrutinized the lateral LLN in the sublingual space by careful palpation. Moreover, extended resection was performed when clinically positive LLNM adhered to the surrounding muscles, mandible, hyoid bone, hypoglossal nerve, or lingual artery.

Patients with ≥ 4 pathological metastatic lymph nodes or ENE with adhesion to the surrounding structures underwent postoperative radiotherapy^21^ of the neck or region of LLNM, with platinum-based anticancer agents administered concurrently, if possible. After radical treatment, patients were followed up every 4 weeks for 1 year, every 2 months for the next year, and every 3 months for another year, with follow-up intervals gradually increasing thereafter. Surveillance included CT or FDG-PET/CT performed 6 months after treatment and every year thereafter. The cervical lymph nodes were carefully monitored by physical examination and ultrasonography. The median follow-up period was 60 months (interquartile range, 27 to 92 months).

### Statistical analyses

Survival was analyzed using the Kaplan–Meier method and compared between groups using the log-rank test. DSS was measured from the date of surgery to the date of death from uncontrolled tongue SCC. Multivariate analyses of factors related to 5y-DSS were performed using the Cox proportional hazards model. The associations between LLNM and categorical variables were assessed using Fisher’s exact test or Pearson’s chi-square test. P < 0.05 was considered statistically significant. All statistical analyses were performed using JMP14 (SAS Institute Inc., Cary, NC, USA).

## Data Availability

The datasets used and analyzed in the current study are available from the corresponding author on reasonable request.

## References

[1] El-Naggar AK, Chan JKC, Grandis JR, Takata T, Slootweg PJ (2017). WHO Classification of Head and Neck Tumours.

[2] Brands MT, Brennan PA, Verbeek ALM, Merkx MAW, Geurts SME (2018). Follow-up after curative treatment for oral squamous cell carcinoma. A critical appraisal of the guidelines and a review of the literature. Eur. J. Surg. Oncol..

[3] Larsen SR, Johansen J, Sørensen JA, Krogdahl A (2009). The prognostic significance of histological features in oral squamous cell carcinoma. J. Oral Pathol. Med..

[4] Oikawa Y (2021). Surgical treatment for oral tongue squamous cell carcinoma: A retrospective study of 432 patients. Glob. Health Med..

[5] Sato J (2018). Hypoxic volume evaluated by 18F-fluoromisonidazole positron emission tomography (FMISO-PET) may be a prognostic factor in patients with oral squamous cell carcinoma: Preliminary analyses. Int. J. Oral Maxillofac. Surg..

[6] Sasahira T, Kirita T, Kuniyasu H (2014). Update of molecular pathobiology in oral cancer: A review. Int. J. Clin. Oncol..

[7] Haagensen C (1972). The Lymphatics in Cancer.

[8] Tomblinson CM, Nagel TH, Hu LS, Zarka MA, Hoxworth JM (2017). Median lingual lymph nodes: Prevalence on imaging and potential implications for oral cavity cancer staging. J. Comput. Assist. Tomogr..

[9] Rouvière H (1938). Anatomy of the Human Lymphatic System.

[10] Ananian SG (2015). Anatomic-histologic study of the floor of the mouth: The lingual lymph nodes. Jpn. J. Clin. Oncol..

[11] Ozeki S, Tashiro H, Okamoto M, Matsushima T (1985). Metastasis to the lingual lymph node in carcinoma of the tongue. J. Maxillofac. Surg..

[12] Han W, Yang X, Huang X, Hu Q, Wang Z (2008). Metastases to lingual lymph nodes from squamous cell carcinoma of the tongue. Br. J. Oral Maxillofac. Surg..

[13] Woolgar JA (1999). Histological distribution of cervical lymph node metastases from intraoral/oropharyngeal squamous cell carcinomas. Br. J. Oral Maxillofac. Surg..

[14] Ando M (2009). Metastatic neck disease beyond the limits of a neck dissection: Attention to the 'para-hyoid' area in T1/2 oral tongue cancer. Jpn. J. Clin. Oncol..

[15] Ando M (2010). Metastases to the lingual nodes in tongue cancer: A pitfall in a conventional neck dissection. Auris Nasus Larynx.

[16] Zhang T, Ord RA, Wei WI, Zhao J (2011). Sublingual lymph node metastasis of early tongue cancer: Report of two cases and review of the literature. Int. J. Oral Maxillofac. Surg..

[17] Jia J, Jia MQ, Zou HX (2018). Lingual lymph nodes in patients with squamous cell carcinoma of the tongue and the floor of the mouth. Head Neck.

[18] Yang W (2020). Lingual lymph node metastasis in cT1-2N0 tongue squamous cell carcinoma: Is it an indicator for elective neck dissection. Front. Oncol..

[19] Fang Q (2019). Value of lingual lymph node metastasis in patients with squamous cell carcinoma of the tongue. Laryngoscope.

[20] Edge S (2009). AJCC Cancer Staging Manual.

[21] Hirai H (2020). Comparison of 50- and 66-Gy total irradiation doses for postoperative cervical treatment of patients with oral squamous cell carcinoma. Oral Oncol..

